# Aortic size, distensibility, and pulse wave velocity changes with aging: longitudinal analysis from Multi-Ethnic Study of Atherosclerosis (MESA)

**DOI:** 10.1186/1532-429X-14-S1-P126

**Published:** 2012-02-01

**Authors:** Chia-Ying Liu, Doris Chen, Gisela Teixido-Tura, Atul R Chugh, Alban Redheuil, Antoinette S Gomes, Martin R Prince, William Hundley, David A Bluemke, Joao A Lima

**Affiliations:** 1 Johns Hopkins University, Baltimore, MD, USA; 2 Department of Cardiology, Hospital General Universitari Vall dHebron, Barcelona, Spain; 3 European Hospital Georges Pompidou APHP, University of Paris Descartes and INSERM U678, Paris, France; 4 Department of Radiology, University of California Los Angeles, Los Angeles, CA, USA; 5 Department of Radiology, Weill Medical College of Cornell University, New York, NY, USA; 6 Division of Cardiology, Deparment of Internal Medicine, Wake Forest University, Winston-Salem, NC, USA; 7 Radiology and Imaging Sciences, National Institutes of Health, Bethesda, MD, USA

## Summary

Arterial stiffening is related to an intricate interplay between aging and other cardiovascular risk factors. The aortic arch accounts for most of the vascular buffering function and is primarily involved in arterial stiffening. MRI has been used to noninvasively measure strain, distensibility, and pulse wave velocity of the ascending aorta. We report aortic size and stiffness changes over mid to late adulthood in longitudinal comparisons with MRI over a 10-year period in the MESA cohort.

## Background

Changes in the human cardiovascular system are an important aspect of aging. Arterial stiffness, a major determinant of increased systolic pulse pressure, is associated with aging and with the incidence of stroke, ischemic heart disease and heart failure. The proximal aorta accounts for most of the global arterial stiffening and subsequent complications. Aortic stiffness can be described by changes in aortic dimensions such as strain (proportional change of aortic area to the minimum area), as well as, combined with pressure, distensibility (relative dimensional change related to changes in local pressure). Another approach to aortic stiffness is the measurement of pulse wave velocity (PWV). While these changes with advancing age have been established in cross-sectional studies, few longitudinal data exist on the evolution of aortic biomechanical parameters. The aims of this study are to assess the longitudinal changes in central aortic size and stiffness measured with MRI over a 10-year period in the MESA cohort.

## Methods

226 longitudinal studies with aortic imaging by MRI were analyzed. MRI was performed at first in MESA1 (baseline, 2000-2001), and then in MESA5 (ten-year follow-up, 2010-2011) after ten years of follow-up. Both exams used 1.5-T whole-body MRI systems and phase contrast cine gradient echo sequence with ECG gating to evaluate aortic size and stiffness. Aortic sagittal oblique plane with black blood sequence was acquired to position the aortic imaging and allowed for the measurement of the distance between the ascending and descending aorta. Images of the ascending and descending aorta were obtained in the transverse plane at the level of the right pulmonary artery perpendicular to the vessel lumen. Aorta analysis was performed using a validated automated software (ARTFUN. INSERM U678). Aortic size, strain and distensibility were reported in the ascending aorta.

## Results

Table 1 lists diastolic aortic size and stiffness aortic parameters measured in baseline and ten-year follow-up stratified by gender. Aortic area and PWV increased significantly with aging. Strain and distensibility of the ascending aorta were also reduced over time.

**Table 1:** Diastolic aortic size and stiffness aortic parameters measured in baseline and ten-year follow-up

Women (N= 131)	Baseline	Ten-year follow up	Paired test p-value
Diastolic aortic size (cm2)	7.2±1.5	8.1±1.7	< 0.001
Ascending aorta Strain (%)	7.8±3.9	7.2±3.7	0.081
Ascending aorta Distensibility (mmhg-1)	1.5±0.9	1.4±0.9	.177
Aortic arch Pulse wave velocity (m/s)	7.2±2.9	8.3±4.1	< 0.001

Men (N=95 )	Baseline	Ten-year follow up	Paired test p-value

Diastolic aortic size (cm2)	8.6±1.7	10.1±2.0	< 0.001
Ascending aorta Strain (%)	7.4±4.1	6.6±3.6	0.086
Ascending aorta Distensibility (mmhg-1)	1.6±1.0	1.4±1.0	0.191
Aortic arch Pulse wave velocity (m/s)	7.2±3.0	8.2±3.5	0.011

GLOBAL (N=226)	Baseline	Ten-year follow up	Paired test p-value

Diastolic aortic size (cm2)	7.8±1.7	8.9±2.1	< 0.001
Ascending aorta Strain (%)	7.6±4.0	7.0±3.6	0.014
Ascending aorta Distensibility (mmhg-1)	1.5±1.0	1.4±0.9	0.059
Aortic arch Pulse wave velocity (m/s)	7.2±2.9	8.3±3.9	< 0.001

Table 2 displays the baseline values for aortic dimensions and aortic stiffness parameters and their absolute changes during follow-up. Ascending aortic area increased more markedly in men than in women during follow-up whereas stiffness parameters changes over time were similar between men and women.

**Table 2:** The baseline values for aortic dimensions and aortic stiffness parameters and their absolute changes during follow-up

	GLOBAL (N=226)	Women (N=131)	Men (N=95)	p-value
Age (years) at baseline	59.4±8.4	59.2±8.3	59.7±8.6	0.646
Diastolic aortic size (cm2) at baseline	7.8±1.7	7.2±1.5	8.6±1.7	< 0.001
Weight at baseline (kg)	82.5±16.5	78.9±16.9	87.4±14.8	< 0.001
Height at baseline (cm)	168.4±10.1	162.6±7.1	176.5±7.9	< 0.001
Ascending aorta Strain (%) at baseline	7.6±4.0	7.8±3.9	7.4±4.1	0.370
Ascending aorta Distensibility (mmhg-1) at baseline	1.5±1.0	1.5±0.9	1.6±1.0	0.641
Aortic arch Pulse wave velocity (m/s) at baseline	7.2±2.9	7.2±2.9	7.2±3.0	0.892
Size change (cm2)	1.2±1.2	0.9±1.1	1.5±1.3	< 0.001
Strain change (%)	-0.7±4.1	-0.6±3.9	-0.8±4.4	0.728
Distensibility change (mmhg-1)	-0.15±1	-0.1±1	-0.2±1	0.749
Pulse wave velocity change (m/s)	1.0±3.4	1.1±3.5	0.9±3.2	0.604

## Conclusions

We report aortic size and stiffness changes over mid to late adulthood in longitudinal comparisons. Further analyses will reveal the correlates of these alterations in multi-ethnic cohorts.

## Funding

The study is supported by N01 HC 95168, Multi-Ethnic Study of Atherosclerosis (MESA), National Heart, Lung, and Blood Institute (NHLBI).

